# Sagittal abdominal diameter and its socioeconomic correlates: perspective of sex differences

**DOI:** 10.1186/s12889-020-09805-z

**Published:** 2021-03-11

**Authors:** Chang Li, Marcelline Harris, Dennis Tsilimingras, Sophia Z. Liu, Ying Sheng, Xuefeng Liu

**Affiliations:** 1 Department of Systems, Population, and Leadership, University of Michigan School of Nursing, Ann Arbor, MI 48109 USA; 2 Department of Family Medicine, Wayne State University School of Medicine, Detroit, MI 48202 USA; 3 Department of Internal Medicine, University of Michigan School of Medicine, Ann Arbor, MI 48109 USA; 4 School of Nursing, Indiana University, Indianapolis, IN 46202 USA; 5 Frankel Cardiovascular Center, University of Michigan School of Medicine, Ann Arbor, MI 48109 USA

**Keywords:** Sagittal abdominal diameter, Visceral adiposity, Sex, Waist circumference, Abdominal obesity

## Abstract

**Background:**

Sagittal abdominal diameter (SAD) is an anthropometric index associated with visceral adiposity. It remains unclear whether SAD and its socio-economic correlates differ in women and men, which limits the epidemiological and clinical applications of the SAD measurement. The aims of this study are to examine the sex differences in SAD and its socio-economic correlates.

**Methods:**

A complex stratified multistage clustered sampling design was used to select 6975 men and 7079 women aged 18 years or more from the National Health Nutrition and Examination Survey 2011–2016, representative of the US civilian non-institutionalized population. SAD was measured in accordance to the standard protocols using a two-arm abdominal caliper. The sex differences in SAD and its socio-economic correlates were evaluated by performing weighted independent t tests and weighted multiple regression.

**Results:**

SAD was lower in women than in men in the entire sample, as well as in all the subgroups characterized by age, race, birth place, household income, and body mass index except for non-Hispanic blacks and those with household income < $20,000. Adjusted for other characteristics, age, birth place, household income, and body mass index were associated with SAD in both women and men. Black women were associated with higher SAD then white women (*p* < .0001), and Hispanic and Asian men were associated with lower SAD than white men (both *p* < .01). Women born in other countries were more likely to have lower SAD than women born in the US (*p* < .0001), and so were men (*p* = .0118). Both women and men with a household income of <$75,000 had higher SAD than those with an income of over $75,000. The associations of age, race, and household income with SAD differed in women and men.

**Conclusion:**

SAD is lower in women than in men, in the general population as well as in the most socio-economic subgroups. While socio-economic correlates of SAD are similar in women and men, the associations of age, race, and household income with SAD vary across sex.

**Supplementary Information:**

**Supplementary information** accompanies this paper at 10.1186/s12889-020-09805-z.

## Background

Accumulating evidence suggests that visceral adipose tissue is more strongly related to metabolic risk factors than subcutaneous adipose tissue [1, 2]. Waist circumference (WC) does not distinguish visceral from subcutaneous adipose tissue, and hence cannot accurately reflect levels of visceral adipose tissue [3]. Instead, sagittal abdominal diameter (SAD) is a simple inexpensive anthropometric measure of visceral adiposity [3], and in some studies, has been shown more useful than other anthropometric measurements, including WC and body mass index (BMI), in assessing health risk [4, 5]. SAD has beeen linked to increased risks of cardiometabolic disorders [5–7] and mortality [8, 9].

Women are reported to have lower intra-abdominal/visceral adiposity than men while the difference is diminished and not consistently seen in the elderly [10–12]. It remains relatively unknown whether SAD, as a manifest measure of visceral adiposity, differs by sex in the general population and in the subgroups defined by socio-economic characteristics. A few studies have evaluated anthropometric measures (including SAD) by sex [13, 14]; yet small sample size, lack of SAD focus, and lack of assessment of SAD in socio-economic subgroups limit the evaluations of sex difference in SAD. Identification of the sex difference in SAD is crucial for better understanding heath disparities in cardiometabolic outcomes among men and women and improving health care for both sexes.

Socio-economic factors, such as age, sex, and education have been linked to the population distribution of SAD in a descriptive population study in Finland [15]. Another recent study has associated certain socio-economic factors with SAD/height ratio in the US representative population [13]. To date, sex differences in the associations of socio-economic factors with SAD have not been fully investigated. Knowledge of sex-specific socio-economic factors associated with SAD could promote the development of sex specific interventions to prevent obesity-related health disparities.

In this study, we used anthropometric data from the US National Health and Nutrition Examination Survey (NHANES) to address: 1) Sex differences in SAD in the entire population as well as in the subgroups characterized by age, race/ethnicity, education, birth place, and household income; 2) Sex differences in socio-economic correlates of SAD. Considering the important role of sex in cardiovascular diseases [16] and the important role of SAD in obesity-related health risks [5–9], our findings will provide useful insights for investigating cardiometabolic risk separately in men and women.

## Methods

### Data source and study population

Beginning in 1999, continuous NHANES is conducted by the National Center for Health Statistics in the Centers for Disease Control and Prevention. It includes a series of two-year cross-sectional nationally representative survey of US civilian noninstitutionalized population [17, 18]. Each survey consists of interview and examination. For each individual, an interview is completed in his/her home, and a health examination is conduted in a mobile examination center. The interview collects demographic, socioeconomic, dietary, and other questionnaire-related data. The examination collects medical, dental, and physiological, and laboratory data. Using a multistage stratified complex probability sampling design, NHANES oversamples older adults, low-income individuals, and certain racial/ethnic groups; participants were assigned weights to account for their unequal sampling probability and nonresponse. All the participants gave informed consent, and the survey was approved by the National Center for Health Statistics Institutional/Ethics Review Board.

In the present study, the sample of participants were chosen from the three most recent cycles of NHANES conducted in 2011–2016. The choice of cycles was determined by data availability of SAD, the only outcome measure in the present study: 2011–2012 represents the first cycle of collecting SAD measurements, and 2015–2016 is the most recent cycle for which data are available. Participants aged less than 18 years who were interviewed only but not examined, who were pregnant during the examination, and/or who had SAD measurements missed, were excluded from the study based on the following considerations. SAD, similar to BMI and WC, may have different definitions or meanings between children and adults, and the purpose of this study was to examine sex differences in SAD among adults. Participants with ‘interview only’ missed the examination of SAD which was our focus measure in the study. Pregnant women experienced dramatic changes in physiology and body shape, and their normal levels of SAD could not be reflected. After applying the above exclusion criteria, we had 14,054 individuals in the final study sample, including 6975 men and 7079 women.

### Sagittal abdominal diameter

**Fig. 1:**
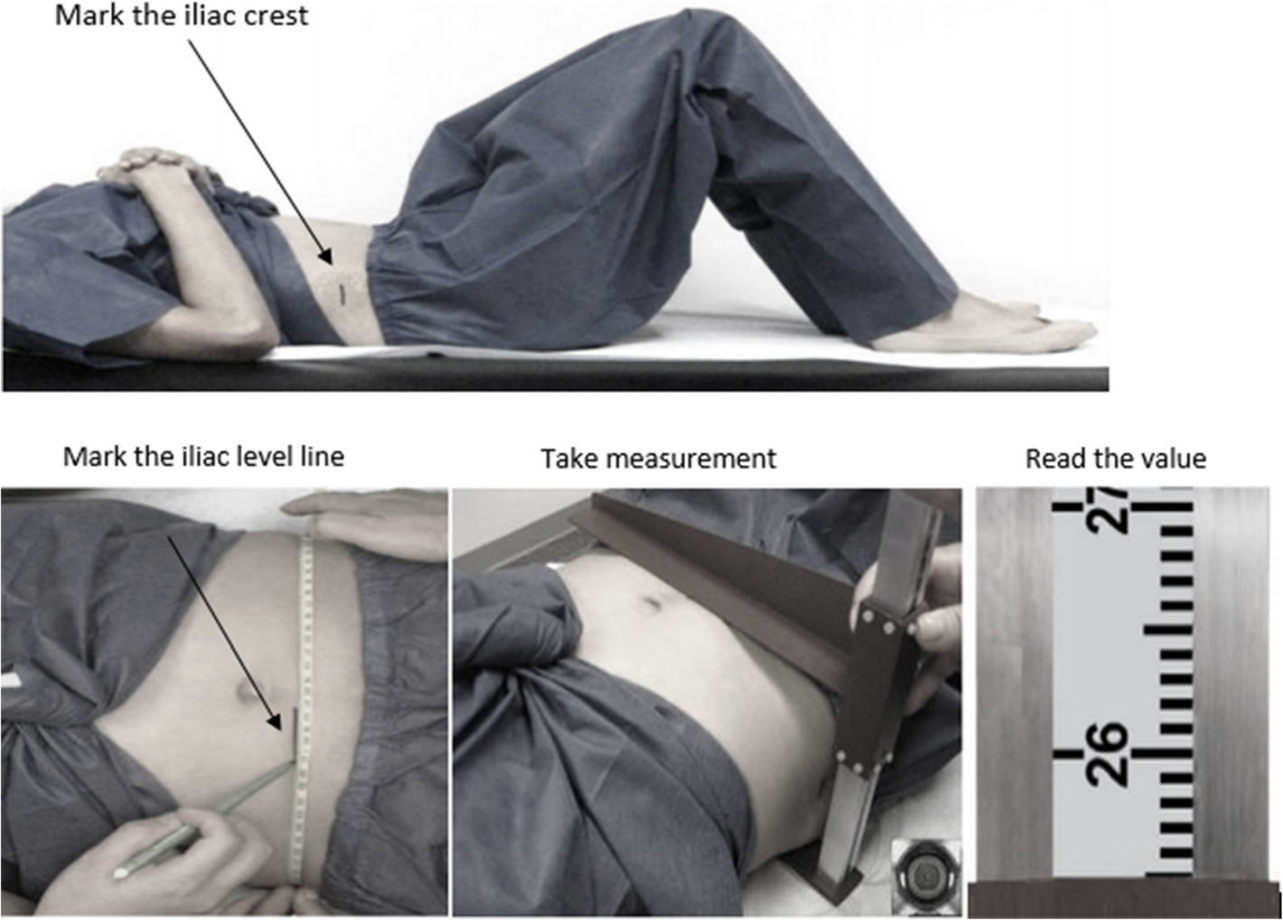
Measurement of sagittal abdominal diameter (cm) by use of a sliding-beam caliper (The images are taken from the National Health and Nutrition Examination Survey: Anthropometry Procedures Manual, 2016 [19])

SAD was measured by a trained examiner when the participant was in the supine position on an examination table (Figure 1) [19]. An abdominal caliper of proper size with lower and upper arms (Holtain Model 609XL, Seritex Inc., NJ, USA) were used to establish the external distance between the front of the abdomen and the small of the back at the iliac level line. The participant was first asked to lie down on the table, bend his or her knees at a 90 degree angle with feet resting flat on the table and arms crossed over the chest. The right and left iliac crests were located, and a line perpendicular to the table on the uppermost lateral border of the right ilium was drawn. A measuring tape was extended over the abdomen without compressing the skin from the left iliac crest to the mark on the right iliac crest. A horizontal line was drawn around 5 cm long, on the abdomen along the iliac level line on the top left edge of the tape.

The caliper’s lower arm was inserted under the small of the back making sure the upper arm exceeds the participant’s abdominal diameter. The shaft of the caliper was adjusted in a vertical position. The caliper’s upper arm was slid down to lightly touch the abdomen with the edge aligned with the iliac level line mark after the participant took in a gentle breath, slowly let the air out and then paused. The measurement was taken when the participant was in the resting phase and the caliper’s shaft was in the vertical position. Up to four SAD readings were taken following the above procedure. The SAD for each person was calculated as an average over the readings.

### Socio-economic characteristics

Socio-economic characteristics considered in this study were all self-reported, including age, sex, race, education, birth place, and household income. They were chosen from a large number of socio-economic variables using model selection techniques and were only significant variables in relation to SAD. Age was categorized as under 30, 30–39, 40–49, 50–59, and 60 years or more. Race/ethnicity included non-Hispanic white, non-Hispanic black, non-Hispanic Asian, Hispanic, and other. The level of education was categorized as high school below, high school graduate/GED or equivalent, and college or above in terms of years in school. Birth place had two categories: born in the US and born in other countries. The family income was grouped into 4 categories: less than $20,000, $20,000–$44,999, $45,000–$74,999, and $75,000 or more. Weight and height were measured using standardized techniques and equipment. BMI was calculated as weight in kilograms divided by the square of height in meters, and then categorized as normal weight (BMI less than 25.0 kg/m^2^), overweight (BMI greater than 25.0 and less than 29.9 kg/m^2^), and obesity (BMI greater than or equal to 30.0 kg/m^2^).

### Statistical analysis

The NHANES supporting and analytical guidelines for surveys 2011–2016 were followed [20]. Stratum, cluster and weight design techniques for survey data were incorporated into data analysis to ensure the representativeness and generalization of the estimates. All the data analyses were performed on PC with windows 10 using survey procedures in SAS version 9.4 (SAS Institute Inc., Cary, NC).

Percentages and standard errors were calculated for categorical variables for men and women to examine sex difference in socio-economic characteristics of subjects. The significance of difference in percentages was tested using weighted χ^2^ tests. To assess unadjusted sex difference in SAD, means and standard errors of SAD were calculated for men and women in each group define by the categories of age, race, education, birth place, household income, and BMI. Weighted independent t tests were used to compare the means of SAD between men and women.

Weighted multiple regression was performed with SAD as a dependent variable and socio-economic characteristics as independent variables including age, sex, race/ethnicity, education, birth place, household income, and BMI. Regression coefficients and standard errors with 95% confidence intervals (CIs) were estimated and adjusted associations of sex with SAD were examined. Wald χ2 tests were useed to examine the significance of parameter estimates. The model was then conducted separately in women and men to identify socio-economic correlates of SAD and the sex differences in associations of the correlates with SAD were test by using weighted independent t tests.

## Results

### Socio-economic characteristics

Average age of the study participants was 47.3 years: 47.4 years for women and 47.2 years for men. From Table 1, we can see that women were 50.4% and men 49.6%. Hispanics were 23.9%, non-Hispanic Asian 12.0%, non-Hispanic blacks 22.3%, and non-Hispanic whites 38.6%. Compared to men, women were more likely to be Hispanic, have a college education or above, live in a poorer household, and be obese.

**Table 1:** Socio-economic characteristics of participants by sex in NHANES 2011–2016

Characteristics	Overall (%)	Sex		
		Women (%)	Men (%)	*p*-value
Age (years)				.0034
Less than 30	20.6 (.34)	19.7 (.47)	21.5 (.49)	
30–39	17.0 (.32)	16.8 (.44)	17.1 (.45)	
40–49	16.8 (.32)	17.8 (.45)	15.8 (.44)	
50–59	16.1 (.31)	16.6 (.44)	15.7 (.44)	
60 or more	29.5 (.38)	29.2 (.54)	29.8 (.55)	
Race/Ethnicity				.015
Hispanic	23.9 (.36)	24.8 (.51)	23.0 (.50)	
Non-Hispanic Asian	12.0 (.42)	12.0 (.39)	12.1 (.39)	
Non-Hispanic Black	22.3 (.35)	22.5 (.50)	22.0 (.50)	
Non-Hispanic White	38.6 (.41)	37.8 (.58)	39.4 (.59)	
Other	3.2 (.15)	2.9 (.20)	3.5 (.22)	
Education				<.0001
Less than 12th grade	22.0 (.35)	20.7 (.48)	23.4 (.51)	
High school graduate/GED or equivalent	22.3 (.35)	20.9 (.48)	23.7 (.51)	
College graduate or above	55.7 (.42)	58.5 (.59)	52.9 (.60)	
Birth place				.63
in US	70.8 (.38)	70.6 (.54)	71.0 (.54)	
in other countries	29.2 (.38)	29.4 (.54)	29.0 (.54)	
Household income (dollars)				.0001
Less than $20,000	21.9 (.35)	23.0 (.50)	20.7 (.49)	
$20,000 to $44,999	29.8 (.39)	30.3 (.55)	29.3 (.55)	
$45,000 to $74,999	19.5 (.33)	19.4 (.47)	19.7 (.48)	
$75,000 or more	28.8 (.38)	27.3 (.53)	30.3 (.55)	
Body mass index (kg/m^2^)				<.0001
Less than 25	31.2 (.39)	32.0 (.55)	30.3 (.55)	
25 to 29	31.7 (.39)	27.0 (.53)	36.5 (.58)	
30 or more	37.1 (.41)	41.0 (.58)	33.3 (.56)	

Note: The numbers in the parentheses are estimated standard errors

### Sex difference in SAD

Overall, SAD was lower in women than in men (22.2 vs. 23.1, *p* < .0001) (Table 2). Analysis of sex difference in SAD by characteristics shows that compared to men, women had lower SAD for all subgroups defined by age, race/ethnicity, education, birth place, household income, and BMI except for non-Hispanic blacks and those with household income less than $20,000 in which men and women have comparable SAD. In addition, Table 2 also shows that non-Hispanic blacks had the highest SAD and non-Hispanic Asians had the lowest SAD among racial/ethnic groups. Adults with household income greater than $75,000 had the lowest SAD and those with income less than $20,000 had the highest SAD among income groups.

**Table 2:** Average sagittal abdominal diameter (cm) by sex among participants in NHANES 2011–2016

Characteristics	Overall	Sex		
		Women	Men	*p*-value
Overall	–	22.2 (.05)	23.1 (.05)	<.0001
Age (years)				
Less than 30	20.6 (.08)	20.2 (.12)	20.9 (.11)	<.0001
30–39	22.3 (.09)	21.6 (.13)	22.9 (.13)	<.0001
40–49	22.9 (.09)	22.4 (.13)	23.5 (.13)	<.0001
50–59	23.6 (.09)	23.3 (.14)	23.9 (.13)	0.0011
60 or more	23.7 (.06)	23.1 (.09)	24.3 (.09)	<.0001
Race/Ethnicity				
Hispanic	22.9 (.07)	22.5 (.10)	23.4 (.10)	<.0001
Non-Hispanic Asian	19.6 (.08)	18.8 (.11)	20.5 (.10)	<.0001
Non-Hispanic Black	23.7 (.08)	23.8 (.11)	23.6 (.12)	.23
Non-Hispanic White	22.8 (.06)	22.1 (.09)	23.6 (.09)	<.0001
Other	22.8 (.23)	22.3 (.35)	23.2 (.31)	.05
Education				
Less than 12th grade	22.9 (.08)	22.7 (.11)	23.0 (.11)	0.028
High school graduate/GED or equivalent	23.0 (.08)	22.7 (.12)	23.3 (.11)	.0002
College graduate or above	22.4 (.05)	21.8 (.07)	23.1 (.07)	<.0001
Birth place				
in US	23.2 (.05)	22.7 (.07)	23.6 (.07)	<.0001
in other countries	21.4 (.06)	20.9 (.09)	22.0 (.08)	<.0001
Household income (dollars)				
Less than $20,000	23.1 (.08)	23.1 (.11)	23.1 (.12)	.92
$20,000 to $44,999	23.1 (.07)	22.8 (.10)	23.3 (.10)	.001
$45,000 to $74,999	22.6 (.09)	22.0 (.12)	23.3 (.12)	<.0001
$75,000 or more	21.9 (.07)	20.9 (.10)	22.9 (.09)	<.0001
Body mass index (kg/m^2^)				
Less than 25	18.2 (.03)	17.6 (.04)	18.9 (.04)	<.0001
25 to 29	22.0 (.03)	21.3 (.05)	22.6 (.04)	<.0001
30 or more	26.9 (.05)	26.4 (.06)	27.7 (.07)	<.0001

Note: The numbers in the parentheses are estimated standard errors

### Trend in SAD over time and age

**Fig. 2:**
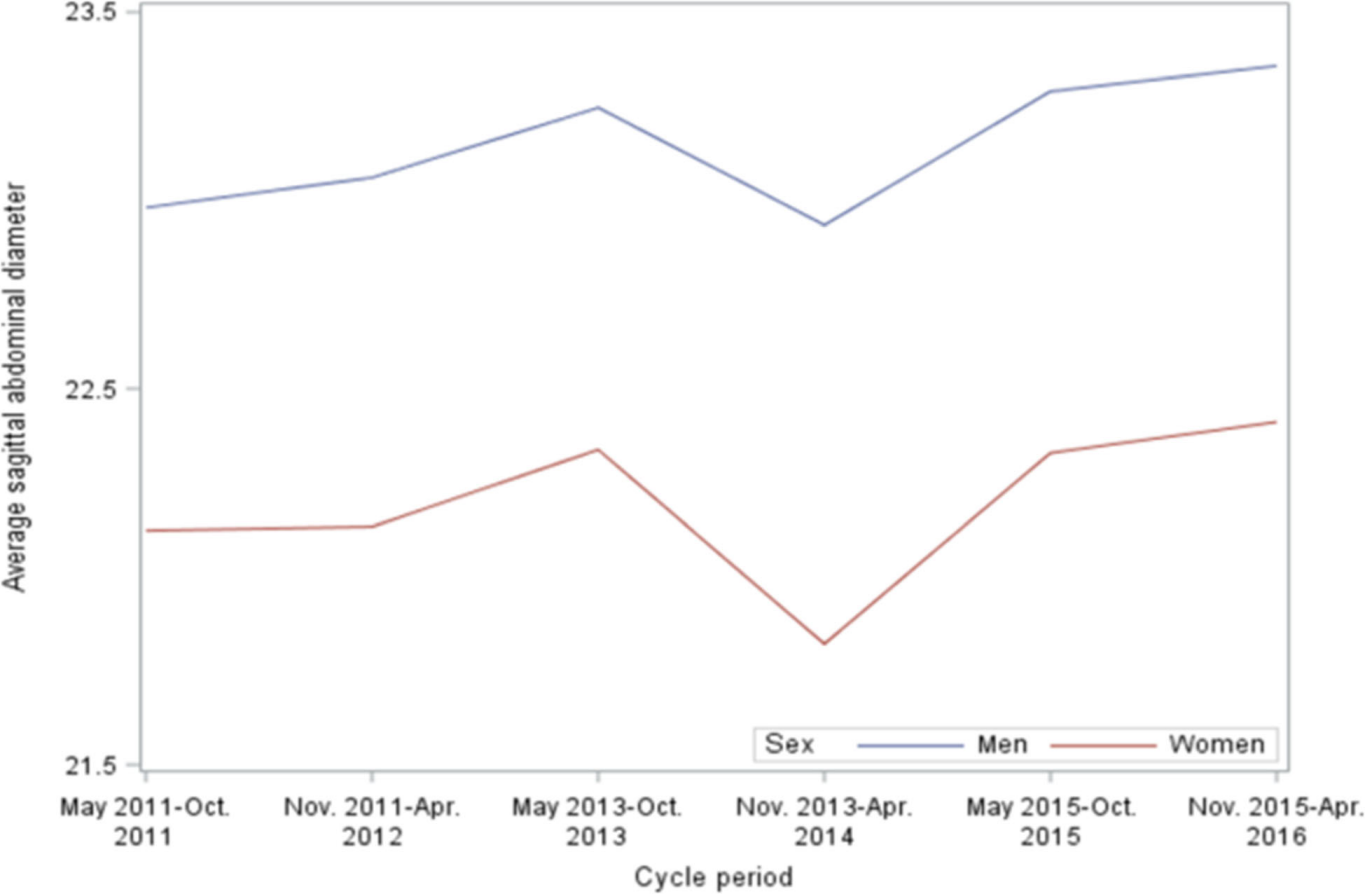
Trend in average sagittal abdominal diameter (cm) over time period in women and men

**Fig. 3:**
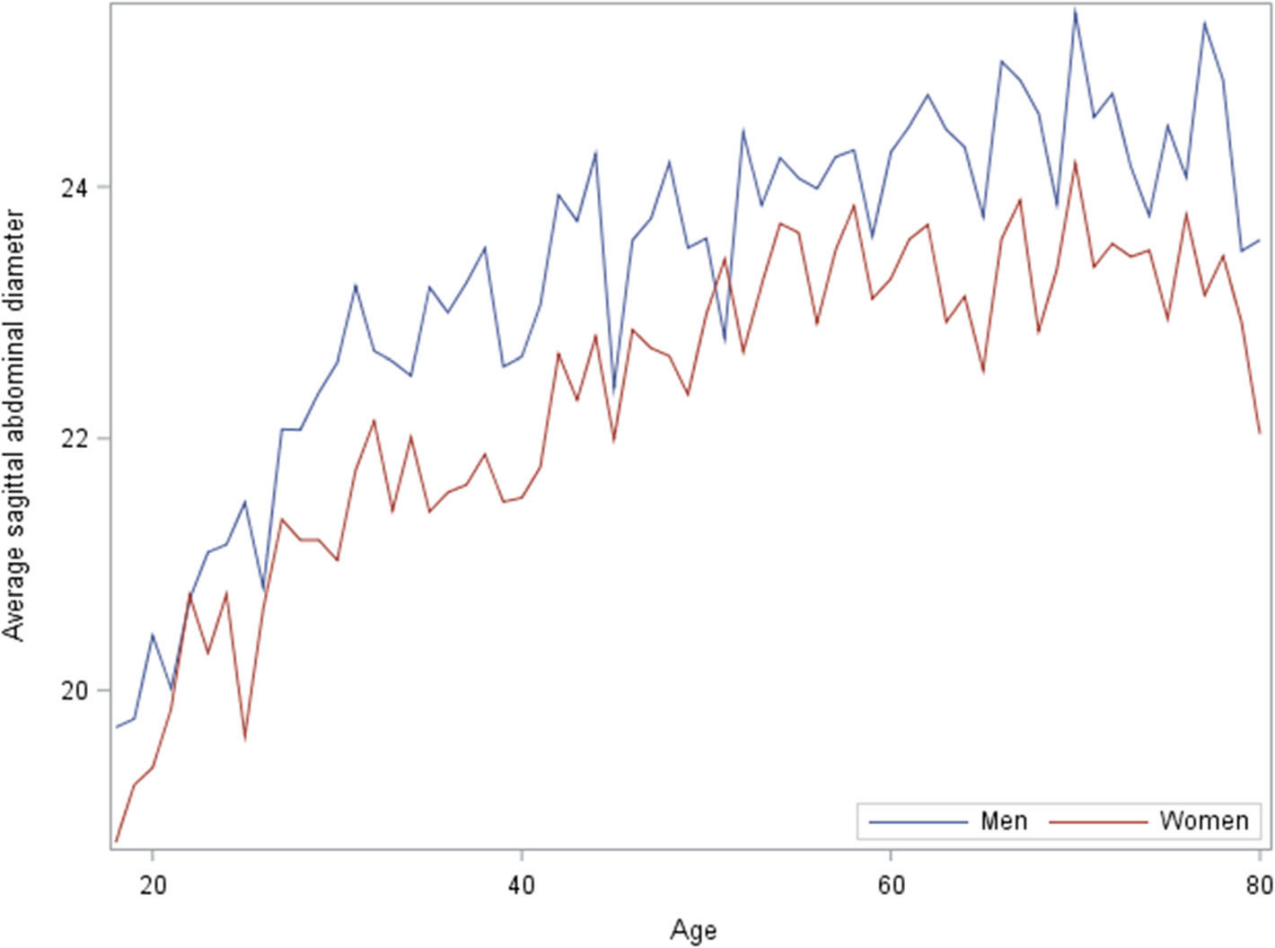
Change of average sagittal abdominal diameter (cm) with age in women and men

SAD was consistently lower in women than in men over the survey period (Figure 2) and age (Figure 3). There were no increasing trends in SAD over the survey period (trend *p* = .23), but there were increasing trends over age (trend *p* < .0001) for both women and men. In both women and men, SAD increased more rapidly at the age of 50 years or below compared to those at the age of > 50 years (Figure 3). The difference in SAD between women and men did not change over age.

### Socio-economic correlates of SAD in women and men

Adjusted for other socioeconomic characteristics, age, sex, race/ethnicity, birth place, household income, and BMI were all associated with SAD (Table 3). Overall, women were 1.3 cm lower than men in average SAD at any given levels of BMI and other variables (*p* < .0001). Hispanics and non-Hispanic Asians had lower SAD than non-Hispanic whites whose SAD was lower than non-Hispanic blacks. Participants born in other countries had lower SAD than those born in the US. Participants with a household income of over $75,000 had lower SAD than those in other income categories of less than $75,000.

**Table 3:** Association of socio-economic factors with sagittal abdominal diameter (cm) in women and men

Characteristics	Overall			Women			Men			*p*-value for sex difference
	Estimate	95% CL	*p*-value	Estimate	95% CL	*p*-value	Estimate	95% CL	*p*-value	
Age (years, vs. < 30)										
30–39	0.8 (.07)	0.7, 0.9	<.0001	0.7 (.10)	0.5, 0.9	<.0001	1.0 (.09)	0.8, 1.2	<.0001	.0028
40–49	1.3 (.07)	1.1, 1.4	<.0001	1.1 (.10)	0.9, 1.3	<.0001	1.4 (.10)	1.2, 1.6	<.0001	.0018
50–59	1.8 (.07)	1.6, 1.9	<.0001	1.6 (.10)	1.4, 1.8	<.0001	1.9 (.10)	1.7, 2.1	<.0001	.0106
60 or more	2.1 (.06)	1.9, 2.2	<.0001	1.8 (.09)	1.6, 1.9	<.0001	2.3 (.08)	2.2, 2.5	<.0001	<.0001
Sex										
Women	−1.3 (.04)	−1.4, −1.2	<.0001	–	–	–	–	–	–	
Race/Ethnicity (vs. Non-Hispanic white)										
Hispanic	−0.2 (.07)	−0.3, −0.1	.0037	−0.0 (.09)	−0.2, 0.1	.66	−0.3 (.09)	−0.5, − 0.2	.0003	.0101
Non-Hispanic Asian	− 0.2 (.09)	− 0.4, − 0.1	.006	− 0.1 (.13)	− 0.4, 0.1	.26	− 0.3 (.12)	− 0.6, − 0.1	.0063	.60
Non-Hispanic black	0.3 (.06)	0.2, 0.4	<.0001	0.5 (.08)	0.4, 0.7	<.0001	0.0 (.08)	−0.1, 0.2	.63	<.0001
Other Race	0.1 (.12)	−0.1, 0.3	.30	0.3 (.18)	−0.1, 0.7	.11	−0.0 (.16)	−0.3, 0.3	.99	.14
Education (vs. College or above)										
Less than 12th grade	−0.0 (.06)	−0.2, 0.1	.54	0.0 (.09)	−0.1, 0.2	.71	−0.1 (.08)	−0.2, 0.1	.40	.025
High school or equivalent	0.0 (.05)	−0.1, 0.1	.76	0.1 (.08)	−0.1, 0.2	.26	−0.0 (.07)	−0.2, 0.1	.61	.0224
Birth place (vs. in the US)										
In other countries	−0.6 (.06)	−0.7, − 0.5	<.0001	−0.7 (.09)	− 0.9, − 0.5	<.0001	−0.5 (.09)	− 0.7, − 0.4	<.0001	.35
Household income (vs. $75,000 or above)										
Under $20,000	0.5 (.06)	0.4, 0.7	<.0001	0.8 (.09)	0.6, 1.0	<.0001	0.3 (.09)	0.1, 0.5	.0006	.0021
$20,000–$44,999	0.5 (.06)	0.4, 0.6	<.0001	0.6 (.08)	0.5, 0.8	<.0001	0.4 (.08)	0.2, 0.5	<.0001	<.0001
$45,000- $74,999	0.3 (.06)	0.1, 0.4	<.0001	0.2 (.09)	0.1, 0.4	.0071	0.3 (.09)	0.1, 0.5	.0009	.002
Body mass index (kg/m^2^, vs. < 25)										
25 to 29	3.3 (.05)	3.2, 3.4	<.0001	3.3 (.08)	3.1, 3.4	<.0001	3.3 (.07)	3.2, 3.5	<.0001	.38
30 or more	8.3 (.05)	8.2, 8.4	<.0001	8.2 (.07)	8.0, 8.3	<.0001	8.4 (.08)	8.2, 8.6	<.0001	.07

Note: The numbers in the parentheses are estimated standard errors

Stratification analysis by sex shows that adjusted for other characteristics, age, birth place, household income, and body mass index were associated with SAD in both women and men (Table 3). Older age groups were associated with higher SAD compared to the group of age 18–29 in both women and men, and the sex differences in age-related associations with SAD are all significant. While both women and men born in other countries had lower SAD than the peers born in the US, the difference in the association of birth place with SAD is not significant between men and women. Income below $2000, between $2000 and 45,000, and/or between $45,000 and $75,000 were associated with higher SAD than peers with income over $75,000 in both women and men, and the sex differences in the associations with SAD were significant (*p* = .0021, *p* < .00001, and *p* = .002, respectively). Compared to non-Hispanic white, Non-Hispanic black was associated with higher SAD in women, and Hispanic and non-Hispanic Asian were associated with lower SAD in men. The associations of Hispanic and non-Hispanic black (vs. non-Hispanic white) with SAD differed across sex (*p* = .0101 and *p* < .0001, respectively).

## Discussion

Our results show that SAD was lower in women than in men in the overall population, as well as in the majority of subgroups defined by age, race/ethnicity, birth place, BMI, and household income. For example, in each BMI category (normal weight, overweight, and obesity), women tended to have an approximate 1.3 cm lower mean SAD than men, and the sex difference in SAD did not change over BMI categories (Table 2). In addition, women had a 0.7 cm, 1.3 cm, 1.1 cm, 0.6 cm, and 1.2 cm lower mean SAD than men in the groups aged< 30, 30 to < 40, 40 to < 50, 50 to < 60, and ≥ 60 years, respectively. SAD is a manifest measure of visceral adipose tissues. The sex difference in SAD may be attributed to the observation that women have more abdominal subcutaneous adipose tissue but less visceral adipose tissue compared to men [21, 22].

BMI, WC and SAD are three measures of obesity and are highly correlated (Supplemental Table 1). While BMI is typically used to measure the general obesity, WC is a measure of abdominal obesity, and measures both subcutaneous and visceral adipose tissues [3]. By examining results from diverse studies of WC after matching the age ranges [23–26], we found that WC was lower in women than in men and the change patterns in WC was similar to the patterns in SAD in this study for women and men. WC is widely used to define abdominal obesity with differential reference ranges/cut points for women and men (88 cm for women and 102 cm for men). Similar differential patterns in SAD across sex groups from our study provide evidence for consideration of sex-specific cut points of SAD when assessing the obesity-related health risk by use of SAD.

Although women have a lower mean WC than men, data from two prospective cohort studies of the Health Professionals Follow-up Study and the Nurses’ Health Study showed that WC predicted the adjusted relative risk of coronary heart disease in both women and men, and the correlation between WC and heart disease was even stronger in women than in men [27]. A different study followed up half a million men and women aged 40–69 years in the United Kingdom and showed that women with bigger waists and waist-to-hip ratios faced a greater excess risk of heart attack than men who had a similar ‘apple shape’ [28]. SAD was thought to have stronger associations with cardio-metabolic disorders than WC [5, 29]. In our study, women tended to have a lower SAD than men, similarly to WC, in the overall population, as well as the subgroups determined by age, race, education, etc. However, it remains unclear whether there is a sex difference in the associations of SAD with cardio-metabolic risk, and further study is needed to examine if the association is stronger in women than in men as WC showed in the previous reports. The strong health risk associations of SAD and WC would indicate that more intensive screening for the risk of cardiovascular disease might help prevent the onset of disease in individuals with an apple shape, especially in women.

SAD increased with age, and the trends in men and women were similar with respect to age. The results can be partly explained by the reports on visceral fat change over age in the previous studies [22, 30]. The process of aging was associated with substantial fat redistribution among depots [31]. Redistribution of fat from subcutaneous to visceral depots was observed from late middle age until the ninth decade of life. Our results also show that the mean SAD increase was faster among younger adults aged< 50 years compared to older ones aged> 50 years for both women and men (Figure 3). The change in SAD over age in our study is similar to WC [22], implying that body fat (both subcutaneous and visceral fat) is mostly accumulated in the first half of the life even though it continues until an older age. Both SAD [5–7] and WC [32] have been associated with cardiometabolic risk which is the leading cause of death in the US and worldwide. The prevention of excess fat in the early life could play a critical role in preventing or delaying obesity-related cardiovascular risk for both women and men.

Although mean BMI and WC among women and men increase in trends for the last two decades, the trends have leveled off in recent years after 2010 [33]. In this study, increasing trends in SAD were not significant for both women and men in 2011–2016, indicating no overall SAD increase over time. This is consistent with trends in BMI and WC, implying that the increases in anthropometric measures of obesity reach a plateau. Numerous obesity prevention and education programs may play an important role in reducing the increasing trend.

In this study, socio-economic correlates of SAD were similar in women and men. Older age, higher BMI, born in the US, and lower household income were all associated with higher SAD in both women and men. However, race/ethnicity groups were differently associated with SAD. Compared to non-Hispanic whites, being Hispanic and non-Hispanic Asian was associated with lower SAD in men but not in women; being non-Hispanic black was associated with higher SAD in women but not in men. A study of subjects, including 66 African American, 72 Hispanic, and 47 white men and women, showed that middle-aged and older African-American men and women had lower visceral fat than Hispanic and white peers. The reports are controversial to our results about racial/ethnic difference in SAD that measures levels of visceral fat. We conducted an analysis using a nationally representative sample of 14,054 individuals, including 3134 non-Hispanic blacks, 3359 Hispanics, and 5425 non-Hispanic whites, and therefore, our data had more power to reflect the difference in visceral fat among racial/ethnic groups.

NHNAES is a national survey of the US civilian non-institutionalized population using a complex stratified multi-stage sampling design. By incorporating into data analysis the features of NHANES design including sampling weights, selection probabilities, and geographic clustering, our results can reasonably be generalized to the entire US non-institutionalized population and the socioeconomic subpopulations of men and women. Another strength of our study is the large sample size that ensures the analysis power for robust and unbiased estimates when comparing the difference in SAD between women and men across socio-economic subgroups. There are several limitations in this study. NHANES is a cross-sectional survey, and the associations of socio-economic factors with SAD could not be interpreted as causal effects. SAD was measured by a two-arm sliding-beam caliper with the possibility of certain levels of measurement errors in SAD. However, trained examiners recorded 4 repeated SAD readings that could minimize the measurement errors. Certain other factors not considered in this study could confound the results, as we focused on sex difference in SAD and its correlates.

## Conclusions

In summary, women had lower SAD than men not only in the overall population, but in the majority of subgroups determined by socio-economic factors, such as age, race, birth place and household income. Given that increased sagittal diameter is linked to intra-abdominal fatness and cardiometabolic risk in both men and women [5, 7], it would be recommended that reference ranges or cutoffs of SAD should be lower in women than in men when using SAD to assess the obesity-related health risk in clinical application and practice. SAD increased with age and increased more rapidly before age of 50 years than after age of 50 years in both women and men, implying that on the average, more amount of visceral fat gain was detected in younger age (< 50 years) than older age (≥50 years). The findings inform the development of education, exercise, and/or diet based programs that target younger populations for control of rapid visceral fat accumulation and for prevention of health risk in the later life. The racial/ethnic difference in SAD (typically, Asian and Hispanic <non-Hispanic white<non-Hispanic black) provide some evidence for adopting different cut points across racial groups for defining the SAD-related obesity, yet further study with focus on racial/ethnic minority groups needs to be investigated. Analysis of SAD correlates indicates that women and men may share the same group of socio-economic factors in relation to SAD.

## Supplementary Information

**Additional file 1: Supplemental table 1**. Correlations between BMI, WC, and SAD.

## Data Availability

Final analysis data will be available to the researchers upon request via email at liuxf@umich.edu or by mail to Dr. Xuefeng Liu, Department of Department of Systems, Populations and Leadership, University of Michigan, 400 North Ingalls Building, Ann Arbor, MI 48109. The general data can be found by clicking on the link https://wwwn.cdc.gov/nchs/nhanes/Default.aspx.

## Abbreviations

BMI: Body mass index
CI: Confidence interval
NHANES: National Health and Nutrition Examination Survey
SAD: Sagittal abdominal diameter
WC: Waist circumference
