# Supplementary material for: Sagittal abdominal diameter and its socioeconomic correlates: perspective of sex differences
Source: BMC Public Health. 2021 Mar 11;21:486. doi: 10.1186/s12889-020-09805-z (PMC7953618; doi:10.1186/s12889-020-09805-z)
Supplement: Supplementary file 1 — Additional file 1: Supplemental table 1. Correlations between BMI, WC, and SAD. [file 12889_2020_9805_MOESM1_ESM.docx]

Supplemental table 1. Correlations between BMI, WC, and SAD

|  | Overall | | | Women | | | Men | | |
| --- | --- | --- | --- | --- | --- | --- | --- | --- | --- |
|  | BMI | WC | SAD | BMI | WC | SAD | BMI | WC | SAD |
| BMI | 1.00 | 0.91 | 0.89 | 1.00 | 0.93 | 0.91 | 1.00 | 0.92 | 0.90 |
| WC | 0.91 | 1.00 | 0.95 | 0.93 | 1.00 | 0.94 | 0.92 | 1.00 | 0.95 |
| SAD | 0.89 | 0.95 | 1.00 | 0.91 | 0.94 | 1.00 | 0.90 | 0.95 | 1.00 |

Note: BMI, body mass index; SAD, sagittal abdominal diameter; WC, waist circumference.
